# Weight Management in Patients with Type 2 Diabetes: a Multidisciplinary Real-world Approach

**DOI:** 10.1007/s11892-018-1030-4

**Published:** 2018-07-17

**Authors:** Osama Hamdy, Sahar Ashrafzadeh, Adham Mottalib

**Affiliations:** Joslin Diabetes Center, Harvard Medical School, One Joslin Place, Boston, MA 02215 USA

**Keywords:** Type 2 diabetes, Obesity, Weight loss, Nutrition therapy, Behavioral therapy, Bariatric surgery

## Abstract

**Purpose of Review:**

Obesity and type 2 diabetes (T2D) are closely linked metabolic diseases. Most individuals with T2D are overweight or obese, which raises their cardiovascular risk. The etiology of both diseases is multifaceted, thus requiring a multidisciplinary approach to control them. This review describes the most effective multidisciplinary approach to weight management in patients with T2D in real-world clinical practice.

**Recent Findings:**

Weight management programs in real-world clinical settings lead to long-term weight loss for up to 5 years.

**Summary:**

Multidisciplinary approach to manage obesity and T2D through weight reduction is feasible in real-world clinical practice and is recommended as part of the treatment plan for patients with T2D who are overweight or obese. Recent data demonstrates that multidisciplinary approach to weight management in patients with T2D results in long-term weight loss and is associated with improved cardiovascular risk factors.

## Introduction

Obesity and type 2 diabetes (T2D) are metabolic diseases that have reached pandemic proportions [1, 2]. The United States Centers for Disease Control and Prevention (CDC) reported that most individuals with T2D are overweight or obese [3]. Furthermore, those afflicted by both T2D and obesity are at greater risk for developing cardiovascular disease [4]. The reasons behind the increased prevalence of obesity and T2D are multifaceted and include genetic, environmental, behavioral, and social elements [5•]. Other epigenetic studies explain the increasing prevalence of T2D from one generation to the next [6].

Controlling both diseases through weight management necessitates an intensive multidisciplinary approach. All clinical guidelines recommend lifestyle modification as the first step in T2D management [7, 8]. However, primary care physicians frequently struggle with lifestyle counseling, which leads to quick initiation of diabetes pharmacotherapy [9–11]. Unfortunately, many of the commonly used antihyperglycemic medications enhance weight gain [12]. As body weight increases, patients become more insulin resistant [13], which further drives the need for higher doses of these medications or adding other antihyperglycemic medications to keep glycemic control at target. This common practice leaves our patients trapped in a vicious cycle.

Conversely, a 7% reduction in body weight achieved through intensive lifestyle intervention was shown to significantly improve insulin sensitivity [14]. Results from the Diabetes Prevention Program (DPP) demonstrated that weight loss over approximately 3 years helped individuals with prediabetes and obesity reduce the incidence of T2D by 58% [15]. The Action for Health in Diabetes (Look AHEAD) study reported that lifestyle modification improved glycemic control in addition to lowering body weight among patients with T2D [16]. Although the study did not achieve the primary endpoints of reducing cardiovascular events and mortality, participants in the intensive lifestyle intervention arm of the study had lower medication use for diabetes, hypertension, and dyslipidemia; had fewer hospitalizations; and showed reduced risk for chronic kidney disease and depression in comparison to the control arm of diabetes support and education [16]. We recently reported that long-term weight loss can be sustained for 5 years in patients with diabetes and obesity who enrolled in an intensive multidisciplinary lifestyle intervention program in real-world clinical practice [17••].

This review describes the components of a practical and effective multidisciplinary approach to weight management for patients with T2D and obesity in real-world clinical practice.

## Multidisciplinary Approach to Weight Management

Multidisciplinary weight management is recommended by medical societies [18]. Data from the National Weight Control Registry show that narrow approaches to weight reduction are rarely effective but that a broader, multifaceted approach is more sustainable [19, 20]. Furthermore, recent studies showed that multidisciplinary weight management results in long-term maintenance of weight loss [17••, 21].

**Fig. 1:**
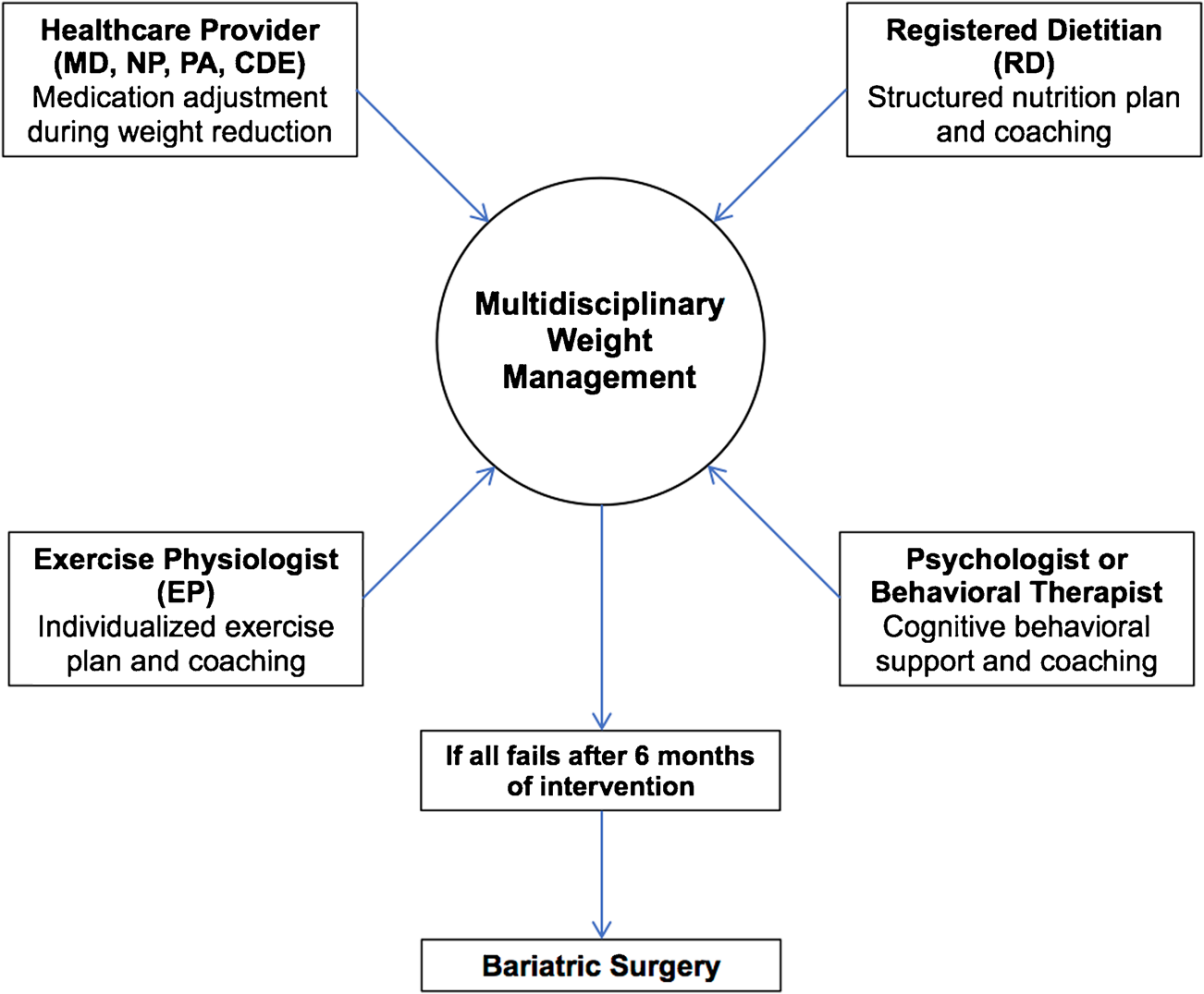
The multidisciplinary approach to weight management in patients with type 2 diabetes and obesity

The Weight Achievement and Intensive Treatment Program (Why WAIT) of the Joslin Diabetes Center is emerging as a viable model of effective multidisciplinary intervention for weight management in real-world clinical practice [22]. The program, which started in 2005, is divided into an initial 12 weeks of intensive intervention during which participants are engaged on a weekly basis for group intervention followed by monthly follow-up sessions to help them maintain weight loss for long term. A recent study showed that participants in the Why WAIT program maintained 6.4% weight loss after 5 years, and participants who achieved ≥ 7% after the first year (53%) maintained 9% weight loss after 5 years [17••]. Other studies on weight management programs in real-world clinical practice have also shown beneficial results over shorter durations [23–25]. The components of the Why WAIT program are described below and are summarized in Fig. 1.

## Components of Multidisciplinary Approach

### Medication Adjustments

Physicians, especially those working in primary care settings, are on the frontline in the fight against obesity and T2D. It is crucial for physicians to not only screen for obesity and diabetes but also to aggressively manage them early in the course of the disease. However, many studies reported that weight management is seldom discussed with patients due to lack of training, time, or reimbursement [26].

Meanwhile, many of the currently used antihyperglycemic medications are known to cause weight gain (e.g., insulins, sulfonylureas, glinides, thiazolidinediones) [12]. For optimal weight reduction through multidisciplinary approach, healthcare providers should reduce, substitute, or even stop, whenever possible, these medications at the beginning of the weight management program. They may be substituted, if applicable, by medications that are weight neutral or by those that enhance weight loss (e.g., metformin, DPP-4 inhibitors, α-glucosidase inhibitors, GLP-1 analogs, SGLT-2 inhibitors, and pramlintide) (Table 1) [12]. Furthermore, use of FDA-approved anti-obesity medications (e.g., lorcaserin, naltrexone/bupropion, topiramate/phentermine, and liraglutide) is encouraged in certain patients with strong appetites [27]. Patients on insulin may be switched to long-acting insulins that induce less weight gain (e.g., insulin detemir, insulin degludec, and insulin glargine U-300) [28–31]. To avoid unnecessary food consumption, patients may be advised to administer short-acting insulin immediately after meals or within 20 min of starting the meal [32]. Two insulins are FDA-approved for postprandial injection: insulin glulisine and fast-acting insulin aspart [33, 34]. With this perspective, patients are educated on injecting insulin based on what was eaten rather than on what they had assumed would be eaten. This technique may reduce mealtime insulin doses and consequently lessen the weight-gaining effect of insulin [32].

**Table 1:** Adjustment of diabetes medication during multidisciplinary weight management in patients with type 2 diabetes and obesity

	List A		List B	
Effect on body weight	Weight gain		Weight neutral	Weight loss
	Significant	Modest		
	Pioglitazone	Sulfonylureas Glimepiride Glipizide XL	Metformin	GLP-1 analogs Exenatide Exenatide ER Liraglutide Albiglutide Dulaglutide Semaglutide
	Sulfonylureas Glyburide Glipizide	Glinides Repaglinide Nateglinide	DPP-4 inhibitors Sitagliptin Saxaglipitin Linagliptin Alogliptin	
	Insulin NPH Glargine Regular Aspart Lispro Glulisine	Insulin Detemir Degludec Glargine U-300 Glulisine (PP) Aspart (PP)	α-Glucosidase inhibitors Acarbose Miglitol	Pramlintide
			Colesevelam	SGLT2-inhibitors Canagliflozin Dapagliflozin Embagliflozin
			Bromocriptine	
Adjustments	Stop, reduce, or switch		Continue	Add

*PP* postprandial

Close monitoring of blood glucose levels is especially important during weight loss. Patients are advised to use a continuous glucose monitor or to check their blood glucose levels 5–8 times daily: before meals, before and after exercise, and at bed time. Blood glucose logs should be reviewed weekly during the intensive period of weight management by healthcare providers including physicians, nurse practitioners, physician assistants, or certified diabetes nurse-educators. Antihyperglycemic medications should be titrated accordingly to prevent hypoglycemia during weight reduction. Occurrence of hypoglycemia with weight loss as the result of improved insulin sensitivity can be a barrier to progressive weight loss and should be avoided even by primitive medication reductions if blood glucose is within target range.

### Nutrition Therapy

Before insulin discovery, dietary intervention was the cornerstone of diabetes management. At that time, clinicians relied solely on carbohydrate restriction within a hypocaloric diet to manage hyperglycemia [35, 36]. However, nutrition therapy was quickly sidelined after the introduction of insulin therapy in 1921. Over the last two decades, supervised nutrition therapy became one of the most effective methods of diabetes and weight management. We recently tested, in a randomized clinical trial, the effects of different models of nutrition therapy on A1C and body weight in patients with T2D and obesity [37]. Our study showed that a structured dietary plan delivered by a registered dietitian (RD) was superior to the currently recommended personalized dietary plan in improving glycemia and body weight [37]. Structured nutrition plans include menus, snack lists, and diabetes-specific formulas. Over 16 weeks of intervention, the use of a structured meal plan, either alone or in combination with weekly phone support by RD, resulted in reduction of A1C by − 0.66% (95% CI − 1.03 to − 0.30) and − 0.61% (95% CI − 1.0 to − 0.23), respectively. It also reduced body weight by − 3.49 kg (95% CI − 4.93 to − 2.05) and − 2.93 kg (95% CI − 4.45 to − 1.42), respectively. In contrast, patients given an individualized meal plan did not show significant change in A1C or body weight from baseline [37].

Proper nutrition therapy usually starts by an RD evaluating potential participants. That evaluation includes review of dietary history and/or 24-h dietary recall and review of adherence to dietary recommendations during previous attempts of weight management. It also includes identification of potential barriers to following a nutrition plan. Each participant should receive a hypocaloric meal plan rounded to the nearest 1200, 1500, or 1800 kcal level for ease of application based on their gender, height, and previous energy intake [22]. In the Look AHEAD study, participants whose weight was over 250 lbs. at baseline were put on a 1500–1800 cal diet plan, and those whose weight was less than 250 lbs. were put on a 1200–1500 cal diet plan [38, 39]. In Why WAIT, men are put on 1800 cal diet plans and women on 1500 cal diet plans. If target weight loss is not achieved within 6 weeks, the dietary plan is advanced to 1500 and 1200 cal, respectively. Women who are shorter than 150 cm are put on 1200 cal diet plans from the beginning of the program. Structured meal plans provide approximately 40–45% of daily energy intake from carbohydrates with 14 g of fiber per 1000 cal, < 35% from fat with < 10% from saturated fat, and 1–1.5 g/kg of adjusted body weight from protein [40, 41]. Effective dietary plans do not calculate protein intake as a percentage of the total calories consumed to avoid unintended reduction in absolute protein intake in a hypocaloric diet. Reduction in absolute protein intake may accelerate lean muscle loss during weight reduction [42]. Minimizing the loss of lean muscle mass during weight management is essential for long-term maintenance of weight loss.

Use of diabetes-specific formulas (DSF) for meal or calorie replacements have been shown to be associated with improved glycemic control and weight reduction [43–46]. Several meal replacement formulas have been tested for weight management [47]. We previously showed that patients with T2D who consumed DSF for breakfast had a lower glucose excursion compared to oatmeal with the same calorie content [48]. Several DSFs were used in both the Look AHEAD study and the Why WAIT program for the initial 6–20 weeks to enhance initial weight reduction [39]. Participants may choose to continue to consume DSFs or to replace them with natural foods after the program.

Participants should also be provided with a list of snack options of 100–200 cal and advised to consume two of them between meals. Their caloric content should be calculated within the prescribed total daily calories. In structured nutrition plans, patients are provided with menus for dinner with details about their ingredients, cooking instructions, and nutrition facts. Participants find it easier to choose a dinner entrée from specified menus rather than calculating calories or following general instructions. Each patient should be provided with a food log, which is reviewed weekly by an RD during the intensive phase of intervention to ensure adherence to the prescribed dietary plan.

### Exercise Intervention

For better long-term results, an exercise physiologist (EP) develops a personalized exercise plan for each patient based on the individual’s age, gender, health status, and exercise capacity. In clinical practice, exercise capacity may be tested by a simple method such as the 6-min walk test [49]. The typical 150 min/week of aerobic exercise or 10,000 steps per day improves fitness but it is not enough for weight reduction or for maintenance of weight loss [50]. Effective exercise intervention for weight management should include a balanced mix of aerobic (endurance) exercise to promote cardiovascular health, resistance (strength) exercise to maintain muscle mass, and flexibility (stretch) exercise to enhance functional capabilities and reduce risk of injury. Exercise plans may progress gradually over 12–24 weeks from 20 min/day for 4 days/week to 60 min/day for 5–6 days/week. After completing the initial intensive phase, participants are usually encouraged to continue to exercise for 60 min/day, 5–6 days/week and maintain ≥ 300 min per week with an emphasis on resistance training to maintain muscle mass. Resistance training is especially important since diabetes is known to worsen sarcopenia (muscle loss that frequently occurs with aging) [51]. Short bouts of exercise of 10 min each distributed during the day were shown to be more sustainable and were associated with similar benefits seen with longer exercise sessions [52]. Use of different exercise methods like circuit and interval training reduce boredom and increase duration of exercises [52, 53]. Exercise is particularly important after the intensive phase of weight management as it helps maintain the weight loss achieved during the intensive period [50].

### Cognitive Behavioral Support

The ability to maintain long-term dietary and exercise modifications relies heavily on patients’ mental and motivational status, which should be addressed through cognitive-behavioral therapy (CBS). Clinical psychologists, behavioral therapists, or social workers are ideal coaches in leading behavioral support sessions, which can be individual or within group settings. Sessions incorporate typical components of CBS, which include behavioral goal setting, self-monitoring of eating and exercise, stimulus control techniques, cognitive restructuring, assertive communication skills, and prevention of relapse [15, 54]. This model was used in the DPP, Look AHEAD study, and Why WAIT program, where it was described in detail [39, 55].

#### Use of Digital Health for Scalable Application of Lifestyle Intervention in Patients with Diabetes

Because of the comprehensive nature of the multidisciplinary approach to weight management, access to such programs may be limited to few patients due to cost or lack of specialized healthcare providers. Mobile phone applications can deliver a diabetes-specific multidisciplinary weight management program at lower cost and with greater accessibility to patients. Currently, over 28,000 smartphone applications focusing on weight management through diet and exercise tracking exist [56], many of which have already demonstrated their capacity to improve body weight and health outcomes in people with diabetes [57, 58]. However, mobile phone applications on the market include, on average, less than 19% of behavioral strategies used in evidence-based lifestyle intervention programs [59]. In particular, strategies for educating patients during their weight management, providing motivational support, reducing stress, and assisting patients with health decision-making have been overlooked [59]. With the introduction of evidence-based design and the integration of blood glucose monitoring systems, mobile health applications present an opportunity for improved accessibility and scalability of weight management interventions.

## Bariatric Surgery

Bariatric surgery is increasingly used for obesity management in patients with diabetes [60]. It should be considered as a valid option for patients with T2D and class 2 and 3 obesity who are unable to reduce their body weight after 6 months of intensive lifestyle intervention. Bariatric surgery, especially Roux-en-Y gastric bypass (RYGB), is a drastic procedure but frequently results in long-term weight loss [61–63]. Studies have shown that among patients with T2D, bariatric surgery improves glycemic control and reduces requirements of antihyperglycemic medications [61–63]. Bariatric surgery may induce partial or complete remission from T2D for several years [64]. The recent and more popular sleeve gastrectomy procedure carries fewer complications than RYGB surgery [65]. A recent study demonstrated a synergistic effect of completing a multidisciplinary weight management program before receiving RYGB compared to receiving RYGB alone [66••]. Serious side events like severe hypoglycemia and severe postural hypotension are not uncommon after RYGB and may require revision of the surgery [67]. A similar procedure called endoscopic sleeve gastroplasty can be done through a gastric endoscopy, eliminating the need for laparoscopic approach [68]. The least effective bariatric surgery for long-term results in patients with T2D is laparoscopic adjustable gastric banding (LAGB) [69]. Over the last few years, US bariatric surgeons started to prefer sleeve gastrectomy over other types of bariatric surgery for its better results and limited complications [65, 70]. Comparison between intensive medical and surgical interventions favored surgery for the magnitude of weight reduction, but the overall quality-of-life measures improved more significantly with non-surgical intervention [71]. Changes in A1C were similar after 1 year between the Why WAIT method of non-surgical intervention and LAGB [71].

## Conclusion

Type 2 diabetes is strongly associated with being overweight or obese. The causes behind both conditions are multifaceted, hence the importance of a multidisciplinary approach to weight management to control both conditions. Recently, a multidisciplinary weight management program was shown to result in long-term weight reduction for 5 years in real-world clinical practice. Intervention methods can be conducted digitally for scalable application to the broader population of patients with T2D and obesity.
